# Perceived Control Buffers Against Variability in Day-to-Day Cognition in Midlife and Older Age

**DOI:** 10.1093/geroni/igaf122.182

**Published:** 2025-12-31

**Authors:** Laura Klepacz, Eric Cerino, Jacqueline Mogle, Jeremy Hamm

**Affiliations:** North Dakota State University, Fargo, North Dakota, United States; Northern Arizona University, Flagstaff, Arizona, United States; RTI Health Solutions, Durham, North Carolina, United States; North Dakota State University, Fargo, North Dakota, United States

## Abstract

Perceived control has been consistently identified as a modifiable predictor of healthy cognitive aging. However, little is known about its link with intraindividual variability in cognitive performance, a sensitive indicator of cognitive aging that confers increased risk of later impairment. A better understanding of the association between perceived control and variability in cognitive performance is needed to elucidate protective psychosocial factors that buffer against heightened variability. Data from the Modifiable Antecedents of Memory and Behavior in Adulthood Study (n = 217, Mage = 53.6 years, SD = 14.8, range = 30-80, 65% female) was used to (a) examine associations between baseline perceived control and day-to-day variability in cognitive performance, and (b) evaluate when in the adult lifespan this relationship becomes most prominent. Multilevel heterogenous variance models revealed that greater perceived control predicted less day-to-day variability (the Level-1 residual variances) in executive functioning (α = -0.23, p < .001) and processing speed (α = -0.25, p < .001), but not episodic memory (α = -0.06, p = .32). Age moderation models showed that higher levels of perceived control were most protective against day-to-day variability in early-to-late midlife. Specifically, perceived control had more prominent negative associations with variability in (a) episodic memory for individuals in their 40s (α = -0.27, p = .057) and (b) processing speed for individuals in their 50s (α = -0.59, p < . 0001). Study findings suggest that perceived control may facilitate the maintenance of more stable cognitive performance, especially among individuals who are in early-to-late midlife.

